# The effectiveness of interventions for reducing stigma related to substance use disorders: a systematic review

**DOI:** 10.1111/j.1360-0443.2011.03601.x

**Published:** 2012-01

**Authors:** James D Livingston, Teresa Milne, Mei Lan Fang, Erica Amari

**Affiliations:** 1 Forensic Psychiatric Services Commission, BC Mental Health and Addiction ServicesPort Coquitlam, British Columbia, Canada; 2 Simon Fraser University, School of CriminologyBurnaby, British Columbia, Canada; 3 Simon Fraser University, Faculty of Health SciencesBurnaby, British Columbia, Canada

**Keywords:** Intervention studies, stigma, substance use disorders, systematic review

## Abstract

**Aims:**

This study provides a systematic review of existing research that has empirically evaluated interventions designed to reduce stigma related to substance use disorders.

**Methods:**

A comprehensive review of electronic databases was conducted to identify evaluations of substance use disorder related stigma interventions. Studies that met inclusion criteria were synthesized and assessed using systematic review methods.

**Results:**

Thirteen studies met the inclusion criteria. The methodological quality of the studies was moderately strong. Interventions of three studies (23%) focused on people with substance use disorders (self-stigma), three studies (23%) targeted the general public (social stigma) and seven studies (54%) focused on medical students and other professional groups (structural stigma). Nine interventions (69%) used approaches that included education and/or direct contact with people who have substance use disorders. All but one study indicated their interventions produced positive effects on at least one stigma outcome measure. None of the interventions have been evaluated across different settings or populations.

**Conclusions:**

A range of interventions demonstrate promise for achieving meaningful improvements in stigma related to substance use disorders. The limited evidence indicates that self-stigma can be reduced through therapeutic interventions such as group-based acceptance and commitment therapy. Effective strategies for addressing social stigma include motivational interviewing and communicating positive stories of people with substance use disorders. For changing stigma at a structural level, contact-based training and education programs targeting medical students and professionals (e.g. police, counsellors) are effective.

## INTRODUCTION

Increasingly, governments and professional organizations are mobilizing resources towards preventing and managing health-related stigma. This coincides with a rapid expansion of research on stigma [1] which, until recently, has concentrated on documenting the magnitude of the problem and understanding its pernicious effects [2–4]. Researchers have been slow to turn their attention towards the question of how stigma associated with mental illness and substance use disorders can be reduced [5,6].

Health-related stigma describes a socio-cultural process in which social groups are devalued, rejected and excluded on the basis of a socially discredited health condition [7]. Stigma may be understood in terms of the different ways it manifests at the self, social and structural levels [8–10]. Self-stigma is defined as a subjective process that is ‘characterized by negative feelings (about self), maladaptive behavior, identity transformation or stereotype endorsement resulting from an individual's experiences, perceptions, or anticipation of negative social reactions’ on the basis of a stigmatized social status or health condition [2]. Social stigma describes ‘the phenomenon of large social groups endorsing stereotypes about and acting against a stigmatized group’[8]. Structural stigma refers to the rules, policies and procedures of institutions that restrict the rights and opportunities for members of stigmatized groups [8,11]. Examples of structural stigma are the negative attitudes and behaviors of representatives of public institutions, such as people who work in the health and criminal justice sectors. Disagreement exists in the literature concerning the levels of stigma, including how many exist and how they are defined. For instance, although including attitudes and behaviors of trainees and professionals within the ‘structural’ level of stigma is consistent with existing definitions and theories [12,13], there are several alternative conceptualizations [9,14,15]. Nevertheless, a three-level framework provides a useful guide for developing strategies aimed at reducing health-related stigma.

### Substance use disorders and stigma

Several studies have found that substance use disorders are more highly stigmatized than other health conditions [16–20]. The relationship between stigma and substance use disorders can manifest differently from that of other stigmatized health conditions, thereby complicating efforts to build social acceptance of people with substance use disorders. Stigma is often used as a tool to discourage and marginalize unhealthy behaviors such as problematic substance use [21–23], which has a collateral consequence of marginalizing and devaluing social groups. Stigmatizing attitudes regarding certain behaviors (e.g. substance use during pregnancy) and groups (e.g. injection drug users) are widely accepted, culturally endorsed and enshrined in policy (e.g. criminal law). Although no empirical evidence exists, some have speculated that reducing the stigma of substance use problems may produce negative effects such as increasing the rates of substance use among younger adolescents [24] and decreasing motivation to seek help among people with substance use problems [25]. Such speculation reflects the broad social discourse surrounding how people with substance use disorders ought to be viewed and treated in our society.

Substance use behaviors are linked symbolically to a range of other stigmatized health conditions [e.g. human immunodeficiency virus/acquired immune deficiency syndrome (HIV/AIDS), hepatitis C virus, mental illness], unsafe behaviors (e.g. impaired driving) and social problems (e.g. poverty, criminality) [18,26–29]. These negative stereotypes guide social action, public policy and the allocation of health-care expenditures. Therefore, people with substance use disorders may experience stigma as a consequence of the culturally endorsed stereotypes that surround the health condition. The fact that stereotypes about substance use disorders have a small degree of accuracy creates challenges for counteracting stigma [30]. The key for anti-stigma interventions is demonstrating that negative attributes (e.g. violence, crime and contagion) are not generally applicable to all members of a particular social group.

Substance use disorders are often treated as a moral and criminal issue, rather than a health concern [18,31]. This is especially true of illegal substances, which are perceived more negatively than legal substances [18,32,33]. Using particular substances (e.g. heroin) has not only been deemed deserving of social disapproval and moral condemnation, but society has also defined such behaviors as crimes. Criminalization of substance-using behaviors exacerbates stigma and produces exclusionary processes that deepen the marginalization of people who use illegal substances [33]. Therefore, the social processes and institutions that are created to control substance use may, in actuality, contribute to its continuance [34].

A final way in which substance use disorders are uniquely related to stigma is that people with this condition are more likely to be perceived as having personal control over their illness and, therefore, are more likely to be held responsible and blamed [20,35,36]. This system of beliefs (i.e. causal attributions) affects the social response to substance use disorders (e.g. anger, avoidance, coercion, punishment) and can influence how people with substance use disorders view themselves [37,38]. Interventions that aim to reduce stigma are likely to be affected adversely by the fact that substance use problems are often perceived as a moral deficit for which a person has corrective control.

### Effects of substance use disorder-related stigma

The detrimental effects of stigma on people with substance use disorders are acute and far-reaching [2–4]. Stigma ascribed to people with substance use disorders exacerbates social alienation [18] and has the potential to impact adversely all domains of life, such as employment, housing and social relationships. Research indicates that stigma contributes to a host of adverse outcomes for people with substance use disorders, including poor mental and physical health [33,39], non-completion of substance use treatment [40], delayed recovery and reintegration processes [22,41,42] and increased involvement in risky behavior (e.g. needle sharing) [43].

Several studies have identified stigma as a significant barrier for accessing health care and substance use treatment services [31,44–47]. Health-care providers may hold negative beliefs about people with substance use disorders, including that they overuse system resources, are not vested in their own health, abuse the system through drug-seeking and diversion and fail to adhere to recommended care [7,48]. Such perceptions can contribute to inequitable and poor provision of care for people with these disorders. As such, individuals with substance use disorders may choose to conceal their substance use problems to avoid stigma, which may result in care provision that does not attend to substance use-related needs (e.g. while pregnant) [33]. The stereotypes associated with substance use treatment services themselves (i.e. methadone maintenance, residential treatment) can also lower the likelihood that people will engage in services [46]. Similarly, health-care providers may refuse to offer certain services (e.g. needle exchange) or may not prescribe effective pharmacological treatments to patients suffering from other illnesses (e.g. cancer, back pain) on account of stigma [49–52].

In Canada, key recommendations from several organizations and agencies [53–55] have focused on addressing the stigma associated with mental health and substance use problems. The purpose of this study was to make available the best possible information by reviewing existing research focused on evaluating the effectiveness of interventions for reducing stigma related to substance use disorders.

## METHODS

Seven electronic databases were selected for their ability to capture relevant literature across disciplines of interest (e.g. medicine, psychology, nursing and social science), including MEDLINE, EMBASE, PsycINFO, CINAHL, Web of Science, EBM Reviews and Cochrane Database of Systematic Reviews. A comprehensive review of these databases was conducted between November and December 2010 to identify English-language published studies, dissertations and conference proceedings. No restrictions were placed on publication year or methodological design. Combinations of keywords related to ‘stigma’ and ‘substance use disorder’ were entered into the above databases, and the ‘titles’ and ‘abstract’ fields were searched (Table S1; details of supporting information are given at the end). Additional publications were identified by scanning reference lists of articles and consulting with experts and key informants. We also hand-searched the content pages of nine journals that had published studies on the topic of substance use-related stigma (Table S2; details of supporting information are given at the end). Grey literature searches were also conducted by entering keywords into search engines, databases and content-relevant websites selected in consultation with experts in the field (Table S3; details of supporting information are given at the end).

### Study selection

Studies were included for full review if they met the following criteria: (i) constituted primary research, (ii) contained an intervention that focused on stigma and substance use disorders, (iii) could be retrieved through university library services or by contacting the author and (iv) were written in English. Reviews and editorials were excluded. No restrictions were made on the publication date or methodological rigor.

The records were divided between two reviewers who read the title and abstract of each record for an evaluation of relevance. To assess inter-rater agreement, approximately 5% of the records (*k* = 315) were selected randomly and rated independently by both reviewers as ‘potentially relevant’ or ‘not relevant’ using the above inclusion criteria. Inter-rater agreement for the title/abstract review was 91.8%. The full-text articles of eligible records rated ‘potentially relevant’ by at least one reviewer (*k* = 160) were obtained, reviewed and rated independently by the two reviewers. Inter-rater agreement for the full-text review was 88.7%. Any discrepancies between the reviewers' ratings were discussed and a consensus was reached.

### Data extraction and quality assessment

A standardized coding form and manual (available from the principal author) were created using an adaptation of Zaza *et al*.'s *Data Abstraction Form for Systematic Reviews*[56]. The coding form also included Downs & Black's *Study Quality Appraisal Checklist*[57], which consists of 27 items and five subscales that assess methodological quality. A higher score indicates better methodological quality. Because of ambiguity in the ‘power’ item, the checklist was modified to assess whether the study authors reported power calculations that indicated an appropriate sample size for detecting clinically important effects (0 = no, 1 = yes). Although the checklist does not have a pre-specified cut-off for acceptable studies, the mid-point score of 14 was used as a guideline to distinguish between low- and high-quality studies.

Training sessions were held in which the research team members thoroughly reviewed the coding form and manual, coded two sample studies, and discussed divergent ratings in detail. The included studies were then reviewed independently and scored by two reviewers. Inter-rater agreement on the quality checklist was 76.9% across the included studies. The two reviewers discussed any discrepancies in ratings and a consensus rating was reached. Where consensus could not be reached on a particular item, a third reviewer provided a rating.

### Data analysis

To gain an understanding of the magnitude of treatment effects, statistical data from each study were converted into individual effect sizes (Hedges' *g*) using the Comprehensive Meta-Analysis version 2 software program (http://www.meta-analysis.com). Substantial clinical, statistical and contextual heterogeneity among the included studies precluded a meta-analysis; therefore, data were synthesized descriptively.

## RESULTS

### Searches

**Figure 1:**
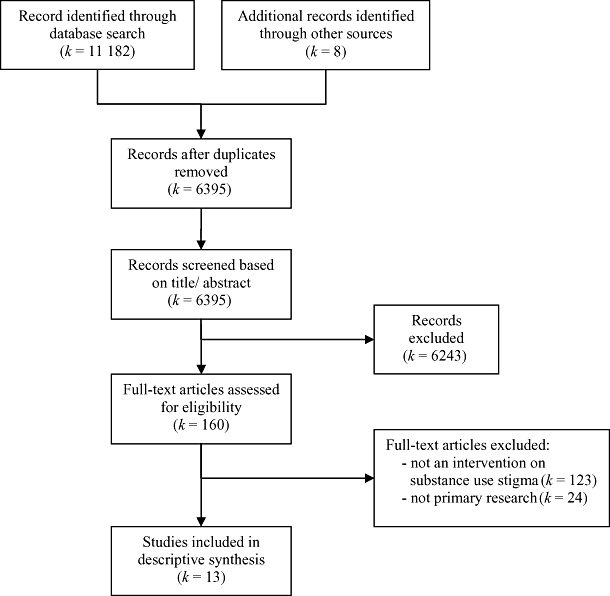
Systematic review search strategy

Using the strategy outlined above, the initial literature search generated a total of 6395 unique records (Fig. 1). Eight records were retrieved from bibliographical searching (*k* = 1), hand-searching journals (*k* = 4) and grey literature searching (*k* = 3). The title/abstract review produced 160 records deemed relevant and eligible for full-text review. Of these, 147 articles were excluded for not containing a substance use-related stigma intervention (*k* = 123) or not involving primary research (*k* = 24). In total, 13 studies met the inclusion criteria for our review [58–70].

### Study characteristics

The majority of studies were conducted in the United States (*k* = 7, 54%), with the remaining studies conducted in the United Kingdom (*k* = 3, 23%), Canada (*k* = 2, 15%) and Australia (*k* = 1, 8%) (Table 1). Most of the studies (*k* = 10, 83%) were published after the year 2000. Because the studies targeted different levels of stigma, the sample sizes varied substantially, ranging from 28 to 445 (median = 108). Eleven studies (85%) used quantitative methodological approaches, including self-report survey methods in 10 (77%) studies. Three studies (23%) assessed stigma beyond the immediate post-intervention period, including a 35-day [60], 90-day [61] and 12-month [67] follow-up. Whereas the majority of studies (*k* = 7, 54%) were not focused on specific types of substances, the remaining interventions focused on alcohol only (*k* = 3, 23%), alcohol and specific drugs (e.g. cocaine) (*k* = 2, 15%) and injection drug use (*k* = 1, 8%). All the studies were restricted to adult populations.

**Table 1:** Characteristics of included studies

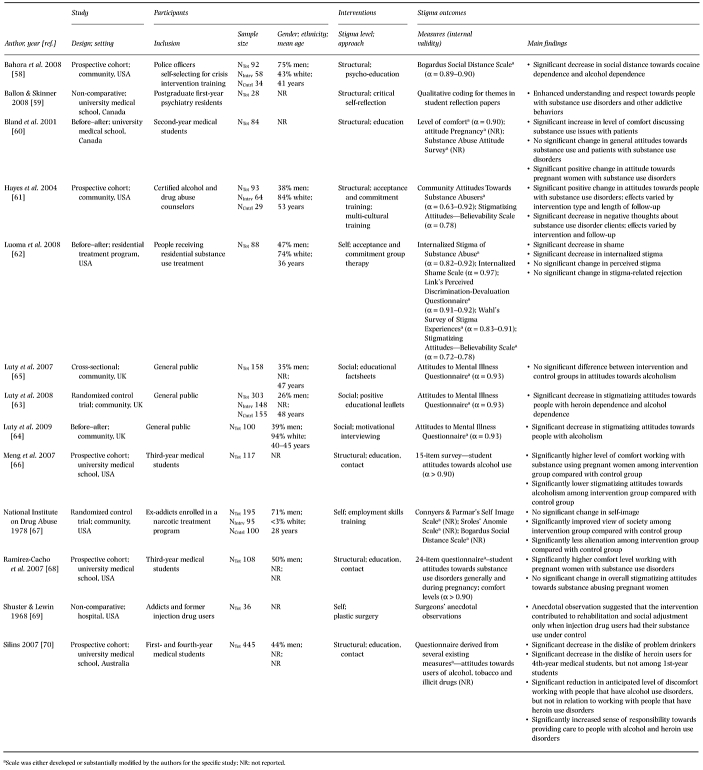

### Methodological quality

All but one included study [67] were published in peer-reviewed journals. Study designs consisted of prospective cohort (*k* = 5, 38%) [58,61,66,68,70], before–after (*k* = 3, 23%) [60,62,64], randomized control trial (*k* = 2, 15%) [63,67], non-comparative (*k* = 2, 15%) [59,69] and cross-sectional (*k* = 1, 8%) [65]. The studies were of variable methodological quality, ranging in overall score from 5 to 19 (maximum = 28) with a mean of 15.8 [standard deviation (SD) = 3.6] (Table 2). Studies with the lowest and highest scores demonstrated 18% and 68% in quality, respectively. Two studies were categorized as low quality [59,69]. The specific methodological problems common across almost all studies were adverse events not measured or reported (*k* = 13), no blinding of assessors (*k* = 13), no blinding of participants (*k* = 12), power calculation not performed or reported (*k* = 12) and unclear representativeness of study participants (*k* = 12).

**Table 2:** Methodological quality of included studies

*Author, year [ref.]*	*Reporting [0–11]*	*External validity [0–3]*	*Bias [0–7]*	*Confounding [0–6]*	*Power [0,1]*	*Overall [0–28]*
Bahora *et al*. 2008 [58]	10	0	5	2	0	17
Ballon & Skinner 2008 [59]	4	3	3	3	0	13
Bland *et al*. 2001 [60]	6	1	5	3	0	15
Hayes *et al*. 2004 [61]	7	1	5	4	0	17
Luoma *et al*. 2008 [62]	10	0	5	2	0	17
Luty *et al*. 2007 [65]	8	2	3	4	1	18
Luty *et al*. 2008 [63]	8	2	5	4	0	19
Luty *et al*. 2009 [64]	8	1	5	4	0	18
Meng *et al*. 2007 [66]	6	2	4	5	0	15
National Institute on Drug Abuse 1978 [67]	7	2	4	3	0	16
Ramirez-Cacho *et al*. 2007 [68]	8	2	4	3	0	17
Shuster & Lewin 1968 [69]	3	0	1	1	0	5
Silins 2007 [70]	8	2	4	4	0	18

### Intervention characteristics

Among the included studies, three interventions (23%) focused on self-stigma, three (23%) targeted social stigma and seven (54%) were categorized as structural stigma interventions. The interventions included a wide range of target groups and methods (Table 1). Nine interventions (69%) used approaches that included education and/or direct contact with people who have substance use disorders.

The self-stigma interventions targeted people with substance use disorders and included an Acceptance and Commitment Therapy (ACT) group [62], a skills training and vocational counseling program [67] and a surgical procedure to remove needle track-marks from injection drug users [69].

The social stigma interventions focused on the general public's attitudes towards substance use disorders. The interventions were comprised of educational factsheets [65], leaflets with photographs depicting positive stories of people with substance use disorders in recovery/ remission [63] and motivational interviewing [64].

The majority of the structural stigma interventions were designed to improve attitudes of medical students towards people with substance use problems, including pregnant women. The approaches used included educational critical reflection techniques [59] and programs comprised of structured education and direct contact with people who have substance use disorders [60,66,68,70]. The remaining two structural stigma interventions targeted attitudes of police officers using a Crisis Intervention Team program [58] and substance use counselors using Acceptance and Commitment Training and multi-cultural training [61].

### Stigma outcome measures

Standardized stigma-related measures were used in 11 (85%) studies; however, most underwent modification to suit the needs of the specific study. The interventions of two studies, which included a qualitative study, were not evaluated using established measures of stigma. The included studies assessed stigma outcomes using a wide variety of instruments (Table 1). For example, two self-stigma studies used standardized measures to assess internalized stigma [62], shame [62], perceived stigma [62], stigma-related rejection [62], self-image [67], social distance [67] and attitudes towards society [67]. The nine studies that evaluated public and structural stigma interventions using established instruments assessed social distance [58], level of comfort [60,66,68] and stigmatizing beliefs and attitudes towards substance use disorders [60,61,63–66,68,70]. Internal reliability was reported for 14 (64%) of the stigma-related outcomes measures included in the studies.

### Intervention outcomes

Of the 13 included studies, more than half (*k* = 7) reported that their interventions achieved positive results on all assessed stigma-related outcomes. Six studies reported mixed results, meaning that they found improvements on some, but not all, stigma variables. The intervention of one study did not result in any significant improvement in substance use-related stigma. Outcomes of various levels of stigma interventions are described below and are summarized in Table 1.

#### Self-stigma

The before–after study by Luoma *et al*. [62] found that group-based ACT resulted in significantly decreased shame [*g* = 1.33, standard error (SE) = 0.35, *P* < 0.001] and internalized stigma (*g* = 1.14, SE = 0.57, *P* < 0.05) among people with substance use disorders. Scores of perceived stigma and stigma-related rejection remained unchanged. The randomized control trial study by the National Institute on Drug Abuse (NIDA) [67] found that employment skills training with people receiving substance use treatment moderately improved participants' view of society (*g* = 0.49, SE = 0.19, *P* < 0.01) and significantly decreased feelings of social alienation (*g* = 0.50, SE = 0.19, *P* < 0.01). The intervention did not lead to significantly improved self-image. Shuster & Lewin's [69] non-comparative, observational study suggested that surgically removing needle track-marks may be beneficial for injection drug users in recovery.

#### Social stigma

A cross-sectional study by Luty *et al*. [65] found that attitudes towards people with substance use disorders (alcoholism) were not significantly different between participants who received didactic educational factsheets and people who did not (*P* > 0.05). Luty *et al*.'s [63] randomized control trial revealed that educational leaflets communicating positive depictions about people with substance use disorders significantly reduced stigmatized attitudes among the general public towards heroin (*g* = 1.50, SE = 0.13, *P* < 0.0001) and alcohol (*g* = 1.25, SE = 0.13, *P* < 0.0001) dependence. Luty *et al*.'s [64] third study found that brief motivational interviews conducted with members of the general public moderately decreased stigmatizing attitudes towards people with alcohol dependence (*g* = 0.44, SE = 0.14, *P* < 0.01).

#### Structural stigma

In relation to improving medical students' attitudes towards substance use disorders, Ballon & Skinner's [59] non-comparison, qualitative study found that incorporating reflection techniques into psychiatry postgraduates training enhanced understanding of the lived experience of substance use disorders and improved clinical skills for working with people who have these conditions. Silins *et al*. [70] examined a structured drug and alcohol education and clinical experience program for medical students, which revealed small but significant decreases in the dislike of problem drinkers among the first-year (*g* = 0.30, SE = 0.10, *P* < 0.01) and fourth-year (*g* = 0.34, SE = 0.11, *P* < 0.01) students. A significant decrease in the dislike of heroin users was achieved only among fourth-year students (*g* = 0.35, SE = 0.11, *P* < 0.01). Among fourth-year students, the intervention also produced moderate and significant reductions in the anticipated level of discomfort in working with people who have alcohol use disorders (*g* = 0.60, SE = 0.12, *P* < 0.001) but not heroin use disorders. Finally, following the intervention, fourth-year students had a significantly increased sense of responsibility towards people with substance use problems, including alcohol (*g* = 0.46, SE = 0.11, *P* < 0.001) and heroin (*g* = 0.36, SE = 0.11, *P* = 0.001).

The three studies that evaluated interventions targeting attitudes towards substance-using pregnant women also found significant improvements among medical students. Bland *et al*.'s [60] before–after evaluation found moderately increased comfort levels discussing substance use issues with patients (*g* = 0.50, SE = 0.17, *P* < 0.01) and moderately improved attitudes specific to pregnant women with substance use disorders (*g* = 0.50, SE = 0.17, *P* < 0.01). There were no significant changes in medical students' attitudes towards substance use generally and people with substance use disorders. Using a prospective cohort design, Meng *et al*. [66] and Ramirez-Cacho *et al*. [68] both found that placing medical students in a specialized prenatal clinic for women with substance use disorders significantly increased their comfort levels in working with this population (*g* = 0.87, SE = 0.20, *P* < 0.001). There were few changes in overall stigmatizing attitudes towards alcoholism in general or towards pregnant women with substance use disorders; however, a small decrease in judgemental feelings towards substance-using pregnant women was detected (*g* = 0.38, SE = 0.20, *P* = 0.05).

Using a prospective cohort design, Bahora *et al*. [58] found that an instructive and interactive crisis intervention skills training program for police significantly reduced officers' desire to maintain social distance from people with substance use disorders, including alcohol (*g* = 1.12, SE = 0.38, *P* < 0.01) and cocaine dependence (*g* = 1.90, SE = 0.45, *P* < 0.001). Hayes *et al*. [61] used a prospective cohort design and found that ACT produced significantly decreased stigmatizing attitudes among substance use counselors at 90-day follow-up (*g* = 0.95, SE = 0.42, *P* < 0.05), but not immediately post-intervention. As well, ACT significantly reduced negative thoughts that substance use counselors held about their clients immediately post-intervention (*g* = 0.85, SE = 0.41, *P* < 0.05) and at 90-day follow-up (*g* = 0.91, SE = 0.41, *P* < 0.05). Substance use counselors who participated in multi-cultural training had significantly decreased stigmatizing attitudes (*g* = 0.72, SE = 0.37, *P* = 0.05) and negative thoughts (*g* = 0.83, SE = 0.38, *P* < 0.05) immediately post-intervention; however, the effects were not sustained at 90-day follow-up.

## DISCUSSION

This systematic review identified a small body of research, comprised of 13 studies, which have empirically evaluated interventions that target stigma related to substance use disorders. Overall, the studies were of moderate research quality, which indicates a risk of bias and confounding that may have affected the cumulative evidence. A major limitation of the included studies is that only three (23%) assessed stigma-related outcomes beyond the immediate post-intervention period. Therefore, the medium- to long-term effects of these interventions remain largely unknown. Another research gap is the absence of substance use-related stigma intervention studies aimed at child and youth populations, which have been identified as important target populations for preventing and reducing stigma [6]. Moreover, research has yet to document whether changes in institutional policies and professional practices actually improve perceptions and experiences of stigma among people with substance use disorders.

The lack of research in this area, as well as the diversity of the interventions within the 13 studies, prevented us from making conclusive remarks concerning the types of interventions that are likely to be effective for reducing self, social and structural stigma related to substance use disorders; however, our review revealed a range of interventions that may be able to influence stigma-related outcomes positively in the context of substance use disorders. Among people with substance use disorders, the limited evidence indicates that therapeutic interventions, such as group-based ACT and vocational counseling, are likely to produce positive effects. This finding is consistent with the broader research literature regarding self-stigma interventions [5,11,15,71]. Improving the attitudes of the general public towards people with substance use disorders may be best accomplished through communication strategies that promote positive stories and through motivational interviewing approaches with particular target groups (e.g. landlords or employers). In contrast, the research suggests that educational factsheets will not achieve meaningful improvements in stigmatizing attitudes among the general public.

Stigma research focused upon other health conditions, such as mental illness and HIV/AIDS, indicates that the effects of education interventions will be enhanced by adding contact-based approaches that facilitate interaction between the public and people who live with stigmatized health conditions. In line with this research, results across several studies included in this review indicated that programs focused on educating medical students about substance use problems and exposing them to people with substance use disorders are likely to decrease their stigmatizing attitudes and increase comfort levels towards working with this population [5,6,15,72,73]. Similarly, interventions that target police officers and substance use counselors have demonstrated positive effects on stigma-related outcomes pertaining to substance use disorders. Furthermore, there is a growing body of research suggesting that interventions can maximize their effectiveness by targeting implicit-automatic processes underlying stigma (e.g. subconscious biases) [74,75]. As such, it would be prudent to integrate this knowledge into anti-stigma interventions.

The methodological limitations of this review must be acknowledged. The first limitation concerns the small number of studies included in our review and the heterogeneity among the studies. Drawing comparisons between studies was made difficult by the fact that they targeted various levels of stigma, employed different measures of stigma and evaluated different types of interventions. Additionally, the studies varied in methodological quality and none received a high score on the *Study Quality Appraisal Checklist*. Secondly, it is possible that relevant studies were not identified. To minimize this possibility, we conducted a comprehensive literature search using several broad search terms across seven electronic databases. As well, our search strategy included a grey literature review and several supplementary methods, which minimized the likelihood that the internal validity of our review was threatened by publication bias and file drawer effects. The final major limitation is that the literature search was restricted to English language publications. Perhaps as a consequence of this inclusion criterion, only English-speaking jurisdictions were represented in the included studies. Consequently, the cross-cultural generalizability of our findings may be restricted.

## CONCLUSION

This review has highlighted a number of interventions and strategies that have demonstrated some success for reducing stigma related to substance use disorders. The findings produced by the 13 included studies require replication, especially as many had small sample sizes, reported mixed results and used uncontrolled study designs. Until such time that there is a more robust body of evidence, it is recommended that these interventions be piloted and evaluated carefully to ascertain whether they are generalizable to different populations and contexts.

## Supporting information

**Table S1** Details of search strategy used for study retrieval.

**Table S2** List of hand-searched journals.

**Table S3** Details of search strategy used for grey literature.
