# Supplementary material for: The effectiveness of interventions for reducing stigma related to substance use disorders: a systematic review
Source: Addiction. 2012 Jan;107(1):39–50. doi: 10.1111/j.1360-0443.2011.03601.x (PMC3272222; doi:10.1111/j.1360-0443.2011.03601.x)
Supplement: Supplementary file 1 [file add0107-0039-SD1.doc]

**Table S1.** *Details of search strategy used for study retrieval*

| Base search terms and combinations |
| --- |
| 1. “substance use disorder*" + (each of ) stigma*, attitude*, discriminat*, inequ*, prejudic*, and sham* 2. substanc* + (each of ) stigma*, attitude*, discriminat*, inequ*, prejudic*, and sham* 3. alcohol* + (each of ) stigma*, attitude*, discriminat*, inequ*, prejudic*, and sham* 4. discrimination + (each of ) "alcohol use*", "alcohol abuse*”, "alcohol dependence" , "alcohol misuse*” 5. addict* + (each of ) stigma*, attitude*, discriminat*, inequ*, prejudic*, and sham* 6. drug* + (each of ) stigma*, attitude*, discriminat*, inequ*, prejudic*, and sham* 7. concurrent + (each of ) stigma*, attitude*, discriminat*, inequ*, prejudic*, and sham* 8. "concurrent disorder*" + (each of ) stigma*, attitude*, discriminat*, inequ*, prejudic*, and sham* 9. "dual use" + (each of ) stigma*, attitude*, discriminat*, inequ*, prejudic*, and sham* 10. "dual dependence" + (each of ) stigma*, attitude*, discriminat*, inequ*, prejudic*, and sham* 11. "dual abuse" + (each of ) stigma*, attitude*, discriminat*, inequ*, prejudic*, and sham* 12. "dual misuse" |

| *Database* | *Search Terms* | *# records retrieved*  *(including duplicates)* | *# records*  *(excluding duplicates within databases)* | *# records*  *(excluding duplicates between databases)* |
| --- | --- | --- | --- | --- |
| Medline | Base search terms plus:  drug* + stigma* + (intervention* or treatment*)  stigma* + (each of) "drug use*", "drug abuse*", "drug dependence", "drug misuse"  drug* + attitude* + (each of) treatment*, intervention*  drug* + attitude* + (intervention* or treatment*)  attitude* + (each of) "drug use*", "drug abuse*", "drug dependence", "drug misuse"  discriminant* + "drug use*", "drug abuse*", "drug dependence", "drug misuse"  sham* + (each of) "drug use*", "drug abuse*", "drug dependence", "drug misuse"  attitude* + (each of ) "substance use*", "substance abuse*", "substance dependence", "substance misuse*"  discrimination + (each of ) "substance use*", "substance abuse*", "substance dependence", "substance misuse*"  alcohol* + attitude* + treatment* or intervention*  alcohol* and discrimination  discrimination + (each of) "alcohol* abuse", "alcohol* dependence", "alcoholism" | 2113 | 1644 | 438 |
| CINAHL | Base search terms plus:  drug* + attitude* + intervention* or treatment*  attitude* + (each of) "drug use*", "drug abuse*", "drug dependence", "drug misuse"  alcohol* + attitude* + treatment* or intervention* | 1303 | 816 | 815 |
| EMBASE | Base search terms plus:  stigma* + (each of) "drug use*", "drug abuse*", "drug dependence", "drug misuse"  attitude* + (each of) "drug use*", "drug abuse*", "drug dependence", "drug misuse"  discriminant* + "drug use*", "drug abuse*", "drug dependence", "drug misuse"  drug combos and inqu*  drug combos and prejudice  drug combos and shame  attitude + (each of ) "substance use*", "substance abuse*", "substance dependence", "substance misuse*"  discrimination + (each of ) "substance use*", "substance abuse*", "substance dependence", "substance misuse*" | 1980 | 1465 | 998 |
| Web of Science | Base search terms plus:  illicit* + substance use disorder* + attitiude*  illicit* + substanc* + stigma*  illicit* + substance use* + attitude*  behavio* + substance abuse* + discriminat*  illicit* + substanc* + sham*  alcohol* + abus* +stigma*  alcohol* + inequ* + abus*  alcohol* + sham* + abus*  drug* + stigma* + abus*  drug* + inequ* + abus*  drug* + sham* + abus*  concurrent + attitude* + disorder*  concurrent + discriminat* + disorder* | 2877 | 2231 | 4 |
| PsychInfo | Base search terms plus:  illicit* + substanc* + stigma*  illicit* + substanc* + discriminat*  illicit* + substanc* + sham*  addict* + sham* + drug*  addict* + sham* + drug*  drug* + abus* + inequ* | 4988 | 3402 | 2560 |
| EBM Reviews | Base search terms plus:  substanc* + attitude* + abus*  alcohol* + attitude* + abus*  drug* + attitude* + abus*  drug* + discriminat* + abus*  drug* + sham* + abus* | 3000 | 1608 | 1565 |
| Cochrane | "substance use disorder$"  alcohol$ and attitude$  alcohol$ and discriminat$  alcohol$ and shame  addict$ and attitude$  addict$ and inequ$  addict$ and sham$  drug$ and stigma$  drug$ and attitude$  drug$ and shame | 18 | 16 | 15 |
