# Supplementary material for: The effectiveness of interventions for reducing stigma related to substance use disorders: a systematic review
Source: Addiction. 2012 Jan;107(1):39–50. doi: 10.1111/j.1360-0443.2011.03601.x (PMC3272222; doi:10.1111/j.1360-0443.2011.03601.x)
Supplement: Supplementary file 2 [file add0107-0039-SD2.doc]

**Table S2.** *List of hand searched journals*

| *Journals* | |
| --- | --- |
| 1. | Addiction (2000-2010) |
| 2. | Addiction Research and Theory (2000-2011) |
| 3. | Addictive Behaviors (2000-2011) |
| 4. | International Journal of Drug Policy (2000-2010) |
| 5. | Journal of Drug Education (2000-2010) |
| 6. | Journal of Substance Abuse Treatment (2000-2011) |
| 7. | Journal of Substance Use (2000-2010) |
| 8. | Substance Abuse (2000-2010) |
| 9. | Substance Use and Misuse (2000-2010) |
