# Supplementary material for: The effectiveness of interventions for reducing stigma related to substance use disorders: a systematic review
Source: Addiction. 2012 Jan;107(1):39–50. doi: 10.1111/j.1360-0443.2011.03601.x (PMC3272222; doi:10.1111/j.1360-0443.2011.03601.x)
Supplement: Supplementary file 3 [file add0107-0039-SD3.doc]

***Table S3.*** *Details of search strategy used for grey literature*

| *Website/database* | *Search Terms* | *# articles retrieved*  *(including duplicates)* | *# unique relevant articles* |
| --- | --- | --- | --- |
| Google | stigma + addiction + intervention + research | 380,000  (28 pages checked) | 1 |
| Alcohol and Drug Abuse Institute Links | stigma  discrimination | 2 | 0 |
| Alcohol Research Group | stigma | 4 | 0 |
| CAMH | stigma + intervention + addiction + results | 214 | 0 |
| Canadian Centre on Substance Abuse | stigma  discrimination | 18 | 0 |
| Centre for Addictions Research of BC | stigma  discrimination | 0 | 0 |
| EAGLE | stigma | 46 | 0 |
| DrugScope | stigma | 26 | 0 |
| European Association of Libraries and Information Services on Alcohol and Other Drugs (ELISAD) | stigma  discrimination | 0 | 0 |
| European Gateway on Alcohol, Drugs, and Addiction | stigma | 3 | 0 |
| European Monitoring Centre for Drugs and Drug Addiction (EMCDDA) | stigma | 15 | 0 |
| Indiana Prevention Resource Center | stigma | 7 | 0 |
| Join Together Online | stigma + reduce | 40 | 1 |
| Mental Health Services Administration | stigma + intervention + research + addiction | 268 | 0 |
| National Drug and Alcohol Research Centre (NDARC) | stigma | 5 | 0 |
| National Center on Addiction and Substance Abuse | stigma | 91 | 0 |
| National Institute on Alcohol Abuse and Alcoholism (NIAAA) | stigma | 28 | 0 |
| National Institute on Drug Abuse | stigma + addiction + reduce  stigma + reduce + research | 214 | 0 |
| National Technical Information Service | stigma + substance  stigma + addiction  discrimination + substance  discrimination + addiction | 31 | 1 |
| Office of National Drug Control Policy | stigma | 12 | 0 |
| SAMHSA's Resource Center | stigma + addiction | 40 | 0 |
| United Nations International Drug Control Programme | stigma + addiction + reduce + research | 99 | 0 |
| WHO Dept. of Mental Health & Substance Abuse | addiction + substance + stigma + reduce + research + intervention + effective | 168 | 0 |
